# Medical Diagnosis with Special Reference to Practical Medicine

**Published:** 1891-01

**Authors:** 


MEDICAL DIAGNOSIS WITH SPECIAL REFERENCE TO PRACTICAL MEDICINE. A guide to the knowledge and discrimination of diseases. By J. M. Da Costa, M. D., L. L. D., Professor of practice of Medicine at the Jefferson Medical College. Philadelphia, Physician to the Pennsylvania Hospital; Consulting Physician to the Children's Hospital, &c. Illustrated with engravings on wood. Seventh edition revised, Philadelphia; J. B. Lippincott Company, 1890.

As professor of practice of medicine and of clinical medicine at the Jefferson Medical College, Philadelphia, the utterances of Dr. Da Costa are entitled to most respectful attention, and that the value of this practical treatise has been appreciated by the profession is proved by the speedy call for a new edition of it. On page 20—general considerations—Dr. Da Costa says, that for a physician to be a thorough diagnostician, "he must be master of something more than of the information supplied to him by semeiology. He must be an anatomist to pronounce with certainty on the seat of the malady; a physiologist to appreciate the state of the great centres and the observation of function—above all, he must be a pathologist in the full sense of the term. He must understand the antagonism between diseases—the frequency with which they coexist, the influence of remedial agents on them; and be cognzant of their natural history and of the general laws governing them,—for how else can he form an estimate of morbid action while in progress?" The work plainly shows on every page that Dr. Da Costa is fully up to the standard, and the requirements which he has so aptly and ably expressed in the above quotation, with the decision born of experience, Professor Da Costa lays down rules of diagnosis which are reliable, and based not only upon experimental knowledge, but upon the observation and experience of years of clinical study. Dr. Da Costa is unquestionably one of the most careful clinical students which this country has produced, and he has enjoyed exceptional facilities for the study of all diseases. From the fact of having had a long period of professional duties. One characteristic of Dr. Da Costa's mind has contributed to make this work of special value: He never strains after that false consistency which makes so many teachers as age creeps over them, cling all the closer to views which they have formed as young men. His mind is unusually receptive and he has kept thoroughly abreast with the current medical opinion of the world. No more fitting monument to the author could be desired than this great work, which has contributed more than any other text book to inculcate in English speaking physicians sound principles in the practice of medicine.—A writer recently reviewing this book, very correctly, says—"it is unnecessary to enter into any detailed notice of a book which has gone through so many editions. The most prominent feature, if one can speak of prominence in connection with symmetry, is that each chapter is so thorough that the specialist in any department of medicine may obtain valuable assistance in the diagnosis of obscure cases."

The book is very neatly bound, and the printing of the text is done with that class of type that makes it a pleasure to read. The profession owe both the author and the publisher a debt of gratitude.

E. V. J.

### AFTER-PAINS.

Dewees is authority for the following rules for the prevention of after-pains:

1. "Do not rupture the membranes before the neck is completely dilated."
2. "After the head is born make no traction, but allow the uterus to expel the shoulders and trunk."
3. "Do not extract the placenta until the womb is thoroughly contracted."
4. "After the placenta is delivered, excite the womb so as to oblige the muscular fibres to contract as much as possible."

Leishman says: "Nothing does so much to prevent their being severe as pressure outside upon the womb during the expulsion of the child and placenta, thereby producing firm contractions." When traction is made upon the cord before the placenta has been expelled from the uterus, the placental vessels are often torn and bleed, and thus a clot is formed. Efforts to deliver the placenta should be directed to producing contractions. These will expel it without leaving a clot; then, by continuing to grasp the womb through the abdominal walls, should it soften, the fact can be recognized, and efforts made to prevent relaxation. This can be done with one hand, and the placenta removed from the vagina with the other.—*Trans. of N. Y. State Society.*

